# Fatigue-Related Changes in Spatiotemporal Parameters, Joint Kinematics and Leg Stiffness in Expert Runners During a Middle-Distance Run

**DOI:** 10.3389/fspor.2021.634258

**Published:** 2021-02-17

**Authors:** Felix Möhler, Cagla Fadillioglu, Thorsten Stein

**Affiliations:** BioMotion Center, Institute of Sports and Sports Science (IfSS), Karlsruhe Institute of Technology, Karlsruhe, Germany

**Keywords:** locomotion, endurance, treadmill, middle-distance, SPM, range of motion, 3D movement analysis

## Abstract

Fatigue with its underlying mechanisms and effects is a broadly discussed topic and an important phenomenon, particularly in endurance sports. Although several studies have already shown a variety of changes in running kinematics with fatigue, few of them have analyzed competitive runners and even fewer have focused on middle-distance running. Furthermore, the studies investigating fatigue-related changes have mostly reported the results in terms of discrete parameters [e.g., range of motion (RoM)] in the frontal or sagittal plane, and therefore potentially overlooked effects occurring in subphases of the gait cycle or in the transverse plane. On this basis, the goal of the present study was to analyze the effects of exhaustive middle-distance running on expert runners by means of both discrete parameters and time series analysis in 3D. In this study, 13 runners ran on a treadmill to voluntary exhaustion at their individually determined fatigue speeds which was held constant during the measurements. Kinematic data were collected by means of a 3D motion capture system. Spatiotemporal and stiffness parameters as well as the RoM of joints and of center of mass (CoM) within the stance and flight phases were calculated. Independent *t*-tests were performed to investigate any changes in means and coefficients of variation (CV) of these parameters between the rested (PRE) and fatigued (POST) state. Statistical parametric mapping method was applied on the time series data of the joints and the CoM. Results from this exploratory study revealed that during a middle-distance run, expert runners change their stance time, rather than their step frequency or step length in order to maintain the constant running speed as long as possible. Increased upper body movements occurred to counteract the increased angular moment of the lower body possibly due to longer stance times. These findings provide insights into adaptation strategies of expert runners during a fatiguing middle-distance run and may serve a valuable information particularly for comparisons with other group of runners (e.g., females or non-athletes) as well with other conditions (e.g., non-constant speed or interval training), and might be useful for the definition of training goals (e.g., functional core training).

## Introduction

Fatigue is a complex phenomenon that develops during both high- and low-intensity exercise, and its origin depends on the intensity and duration of exercise (Millet and Lepers, 2004). Fatigue is therefore inherent in endurance sports, for example in running. Several studies have shown that fatigue causes changes in running kinematics (Winter et al., 2017; Kim et al., 2018), which in turn may decrease performance and increase injury risk (Hreljac et al., 2000). Deeper understanding of fatigue-related changes is therefore essential for optimization of training loads or prevention of injuries.

Most previous studies investigated the influence of fatigue during long-distance runs (>3,000 m or an equivalent time) (Winter et al., 2017; Kim et al., 2018; García-Pinillos et al., 2020; Willwacher et al., 2020), and only a few analyzed biomechanical alterations of competitive-level runners under exhaustive effort. Sanno et al. (2018) compared competitive with recreational runners over a 10 km run and found an increased knee flexion at touchdown in both groups as well as increases in maximal knee flexion and decreases in plantar flexion at toe off in the recreational runners (Sanno et al., 2018). Willwacher et al. (2020) observed kinematic adaptations in both recreational and competitive runners during a 10 km treadmill run in the non-sagittal planes. They reported changes between the pre- and post-fatigue state, particularly in hip adduction, ankle eversion and in knee valgus angle, although they did not consider spatiotemporal parameters or changes in the sagittal plane. García-Pinillos et al. (2020) analyzed spatiotemporal parameters and stiffness changes in trained male endurance runners during a 60 min treadmill run, but did not include any results concerning joint kinematics in their study. They reported an increased contact time and step variability as well as decreased flight time and leg stiffness in fatigued runners.

To date, only a limited number of studies have examined kinematic alterations related to fatigue over middle-distance runs (≤3,000 m or an equivalent time). Rabita et al. (2013) evaluated the changes in spring-mass behavior of runners during an effort with a mean time to exhaustion of 5:53 min. They reported decreased leg stiffness and altered spatiotemporal parameters, although they did not include joint kinematics in their analysis. Derrick et al. (2002) examined kinematic adjustments and their influences on shock attenuation potential during an exhaustive run (average time 15:42 min) of recreational runners by means of mobile sensors, and suggested that kinematic adaptations may lead to increased metabolic cost. A recent study by García-Pinillos et al. (2019) analyzed kinematic adaptations during two high-intensity interval programs using a high-speed camera, and reported no changes in the spatiotemporal and kinematic variables studied. In another study examining joint angle alterations and changes in shock absorption capacity after a brief exhaustive run, no significant differences between pre-and post-fatigue states were found (Abt et al., 2011). Maas et al. (2018) analyzed both experienced and novice runners during a run to exhaustion during a 3,200 m time trial pace using a 3D motion capture system. They reported increases in pelvic tilt, pelvic range of motion (RoM) and knee abduction as well as decreases in hip adduction and ankle plantar flexion. Furthermore, they showed that novice runners exhibit larger kinematic adjustments than experienced runners. Another group of researchers also analyzed novice runners in comparison to experienced runners focusing on stride-to-stride variability (Mo and Chow, 2018a) and coordination variability (Mo and Chow, 2018b) for prolonged treadmill run at anaerobic threshold speed. They reported that novice and experienced runners differ from each other particularly in terms of both stride-to-stride and coordination variability.

Several studies only analyzed motion in 1D or 2D (Winter et al., 2017; Kim et al., 2018), which could limit the scope of the results. As suggested by Willwacher et al. (2020), fatigue may cause alterations in non-sagittal planes. Therefore, analyses should comprise all of the relevant and anatomically-possible degrees of freedom. In addition, including upper body kinematics could improve the explanatory value of results, since upper body rotation has been found to increase with fatigue in long distance runs and was hypothesized to be detrimental for performance and to increase injury risk (Strohrmann et al., 2012). In addition, García-Pinillos et al. (2020) argued that robust conclusions regarding coordination, injury prevention and sports performance depend not only the mean values of spatiotemporal parameters but also their variability, which in their study was operationalized as the coefficient of variation. They reported increased variability with fatigue, whereas Hanley and Tucker (2018) found only moderate changes in variability between successive testing distances in their study. Variability of movement patterns is all in all an important and widely discussed topic in a wide range of disciplines, among others in sports biomechanics, since it helps to understand adaptation strategies as well as flexibility of the motor system in movement production (Meardon et al., 2011; Mo and Chow, 2018a,b). In addition, movement variability is speed-dependent (Meardon et al., 2011), so different running distances may lead to different variability characteristics since running speed changes with running distance. Similarly, the expertise of the runners is a factor influencing movement variability. Accordingly, different groups of participants as well as different study designs may provide different results (Mo and Chow, 2018a,b).

Stiffness is another important biomechanical parameter in analyzes of running gait because of its close relationship to injuries and performance (Butler et al., 2003) as well as to fatigue (Rabita et al., 2013; García-Pinillos et al., 2020), however a clear consensus regarding the relationship between these parameters is still lacking. Butler et al. (2003) reported that increased stiffness may be beneficial to sports performance and decreased stiffness may be associated with soft tissue injuries. On the other hand, Lorimer and Hume (2016) concluded that high lower body stiffness may be associated with Achilles tendon injuries, particularly in association with training on surfaces with low stiffness properties. All in all, leg and vertical stiffness might be an important aspect for performance as well as for injury prevention (Pappas et al., 2015).

In summary, existing studies have used a multitude of fatigue protocols, measurement devices, and dependent variables with participants from a broad range of expertise levels. Accordingly, there is no consensus about the effects of fatigue on the biomechanics of middle-distance running. The goal of the present study was to analyze the possible effects of fatigue on spatiotemporal parameters, leg and vertical stiffness, 3D joint kinematics as well as the center of mass (CoM) trajectory during a middle-distance run by expert runners. In addition, this study aimed to conduct an explorative analysis of entire time series data by means of statistical parametric mapping (SPM) and important discrete parameters (spatiotemporal parameters and RoM). The presented results may provide informative data concerning biomechanical adaptations of competitive-level runners during an exhaustive middle-distance run and may be useful for future research particularly for comparisons with different expertise levels (e.g., non-athletes) or other running distances.

## Materials and Methods

### Data Set

Data from a previously published study (Möhler et al., 2019) were re-analyzed. The participants were 13 male runners (age: 23.5 ± 3.6 years, BMI: 20.6 ± 1.7 kg/m^2^). Inclusion criteria were a 10 km record below 35 min (32:59 ± 01:19 min), a minimum mileage von 50 km/week during the 8 weeks preceding the measurement and an active membership in a running club for at least 2 years (7.2 ± 3.2 years). Exclusion criteria were pain in the lower limbs or recent injuries. All participants provided written informed consent. The study was approved by the ethics committee of the Karlsruhe Institute of Technology. Each participant came to the laboratory on two different days 1 week apart. The tests were performed on a motorized treadmill (h/p/cosmos Saturn, Nussdorf-Traunstein, Germany). For safety reasons, subject wore a safety harness which was connected to an emergency stop. During the first visit, their individual fatigue speed was determined during an incremental lactate threshold test. The test started at 8 km/h, the duration per step was 3 min, there were 30 s of rest between the steps and the increment between the steps was 2 km/h. The individual fatigue speed was determined on the basis of lactate values and by means of the critical power concept developed by Monod and Scherrer (1965). The fatigue speed was defined as the speed that runners were potentially able to run for 10 min at most. This speed was at 110% of their speed at 4 mmol/l lactate (19.27 ± 0.72 km/h). During the second visit, the actual measurement was performed. At first, a standardized treadmill familiarization [6 min of walking, 6 min of running (Matsas et al., 2000; Lavcanska et al., 2005)] was performed. Afterwards, participants ran at their individually determined fatigue speed until voluntary exhaustion, which was reached after 4:06 ± 0:52 min (1.34 ± 0.27 km). Exhaustion was confirmed by a Borg-scale rating (Borg, 1982) of 19.6 ± 0.65. Participants wore their own running shoes. During running, 41 marker trajectories were captured by 11 infrared cameras at a recording frequency of 200 Hz (Vicon Motion Systems; Oxford Metrics Group, Oxford, UK). A total of 19 strides were captured at the beginning of the run (PRE measurement, non-fatigued state) and 19 strides immediately before exhaustion (POST measurement, fatigued state).

### Data Processing

Data were preprocessed using Vicon Nexus software V1.8.5 (Vicon Motion Systems Ltd., UK). All subsequent data processing operations were performed with MATLAB R2020a (MathWorks, Natick, MA, USA). To obtain joint angles, an inverse kinematics calculation was conducted using a modified version of the full-body model Dynamicus (ALASKA) (Härtel and Hermsdorf, 2006). Foot strikes were identified using the vertical speed of the foot markers whereas toe-off was identified using the vertical acceleration (Leitch et al., 2011).

Duration of stance (time between right foot strike and right toe off), duration of flight (right toe off to left foot strike), and stride frequency (right foot strikes per second) were analyzed as spatiotemporal parameters in order to generally characterize the running kinematics of our participants. Vertical stiffness and leg stiffness were also included in the analyses because these parameters may change under neuromuscular fatigue (Dutto and Smith, 2002; García-Pinillos et al., 2020) and therefore be helpful to understand the general adaptation patterns in presence of fatigue, especially in relation to the spatiotemporal changes. Since the measurements were performed on a non-instrumented treadmill, the stiffness parameters were estimated based on kinematic data as suggested by Morin et al. (2005), who showed the validity of this method. For both spatiotemporal and stiffness parameters, the coefficient of variation (CV) was calculated alongside the mean and standard deviation. The CV was included because it may reveal changes in the stability of the coordination pattern (Jordan et al., 2009). Furthermore, there are some studies indicating a relationship between step variability and injuries (Meardon et al., 2011) as well as endurance performance (Nakayama et al., 2010).

Joint kinematics were analyzed for the lower extremities (ankle, knee, and hip joints) and torso (lumbar spine and thoracic spine joints) in the sagittal (S), frontal (F), and transversal (T) planes to incorporate all important degrees of freedom and constraints. Time series data of joints were analyzed by means of SPM because it has been suggested to be superior to over-simplified discrete parameter analyses by being capable of identifying field regions which co-vary significantly with the experimental design (Pataky et al., 2013). As well as analysis of the entire time series, RoM was calculated as the difference between the maximum and the minimum joint angle for both stance (right foot strike to right toe off) and flight phase (right toe off to left foot strike). The RoM results could be helpful for understanding adaptations to fatigue, particularly in terms of injuries, because it literally manifests the limits of motions. Increases in RoM may indicate a higher risk of soft tissue damages because of potentially increased strains in these tissues. Similarly, analysis of the CoM was accomplished by considering both the time series and the RoM.

### Statistics

For the spatiotemporal parameters and the RoM, the 19 PRE strides and the 19 POST strides were averaged for each participant for statistical analysis. The PRE and POST averages were compared using paired *t*-tests and Cohen's *d* was calculated as a measure of effect size. Normality distribution was verified using the Shapiro-Wilk-test. For all statistical tests, the level of significance was set *a priori* to *p* = 0.05. Cohen's *d* was classified as the following: *d* < 0.5 small effect, 0.5 < *d* < 0.8 medium effect and *d* > 0.8 large effect (Cohen, 1992). The joint angle time series were time-normalized and compared using statistical non-parametric mapping (www.spm1d.org) due to non-normal distribution. All analyses were performed for the right side assuming that both legs would fatigue at a similar rate (Pappas et al., 2015).

## Results

### Spatiotemporal Parameters and Their Variability

Aiming at investigating spatiotemporal characteristics both in PRE and POST, stance time, time of flight, stride frequency, and their variability across multiple strides were estimated. The results are represented in Table 1. Analysis of the spatiotemporal parameters revealed a significantly higher stance time (PRE: 0.16 s, POST: 0.17 s, *p* < 0.001, *d* = 3.016) and shorter time of flight (PRE: 0.33 s, POST: 0.31 s, *p* < 0.001, *d* = 2.077). The CV of the spatiotemporal parameters did not show any significant changes (Table 1).

**Table 1:** Spatiotemporal parameters, vertical, and leg stiffness together with corresponding coefficients of variation (CV) shown as mean ± standard deviation.

	**PRE**	**POST**	***P***	***d***
**Stance time [s]**	**0.16** **±** **0.02**	**0.17** **±** **0.02**	**<** **0.001**	**3.016**
**Time of flight [s]**	**0.33** **±** **0.04**	**0.31** **±** **0.03**	**<** **0.001**	**2.077**
Stride frequency [1/s]	1.53 ± 0.07	1.54 ± 0.07	0.120	0.464
**Vertical stiffness [kN/m]**	**20.55** **±** **3.98**	**18.01** **±** **4.56**	**<** **0.001**	**1.701**
**Leg stiffness [kN/m]**	**12.40** **±** **2.62**	**10.56** **±** **2.90**	**<** **0.001**	**1.856**
***Coefficients of variation***				
Stance time	0.03 ± 0.01	0.03 ± 0.01	0.175	0.399
Time of flight	0.02 ± 0.01	0.01 ± 0.07	0.069	0.555
Stride frequency	0.01 ± 0.00	0.01 ± 0.00	0.230	0.351
**Vertical stiffness**	**0.08** **±** **0.02**	**0.06** **±** **0.02**	**0.045**	**0.619**
**Leg stiffness**	**0.08** **±** **0.03**	**0.07** **±** **0.02**	**0.047**	**0.613**

*p-values as calculated by the dependent t-test and Cohen's d as effect sizes are given. Significant differences are highlighted in bold (p < 0.05). Cohen's d effect sizes of <0.50, 0.5–0.8, and >0.8 indicate small, medium, and large effects, respectively*.

### Vertical and Leg Stiffness and Their Variability

Vertical and leg stiffness were included in order to be able to explain changes in spatiotemporal parameters with respect to changes in stiffness, because stiffness is thought to exert a major effect on various athletic variables related to running kinematics (Brughelli and Cronin, 2008). In the POST, both the leg and the vertical stiffness decreased significantly with high effect sizes (PRE_leg_: 12.40 kN/m, POST_leg_: 10.56 kN/m, *p* < 0.001, *d* = 1.856; PRE_vertical_: 20.55 kN/m, POST_vertical_: 18.01 kN/m, *p* < 0.001, *d* = 1.701), which were in accordance with increased stance times. The CV of both stiffness parameters also decreased significantly with medium effect sizes indicating a less variable stiffness over strides in POST (PRE_leg_: 0.08, POST_leg_: 0.07, *p* = 0.047, *d* = 0.613; PRE_vertical_: 0.08, POST_vertical_: 0.06, *p* = 0.045, *d* = 0.619) (Table 1).

### Analyses of Range of Motion

In the stance phase, the RoM predominantly increased with fatigue (Table 2). Both at the ankle and at the knee joint, RoM increased significantly in the sagittal plane with a high effect size (Ankle PRE_S_: 51.15°, POST_S_: 53.55°, *p* < 0.001, *d* = 1.23; Knee PRE_S_: 37.81°, POST_S_: 40.97°, *p* < 0.001, *d* = 1.451). The remaining joints, namely the hip (PRE_S_: 53.55°, POST_S_: 56.87°, *p* < 0.001, *d* = 2.200; PRE_F_: 17.10°, POST_F_: 18.82°, *p* < 0.001, *d* = 1.282; PRE_T_: 9.39°, POST_T_: 11.86°, *p* < 0.001, *d* = 1.442), the lumbar spine (PRE_F_: 8.10°, POST_F_ 10.05°, *p* < 0.001, *d* = 1.513, PRE_T_: 3.78°, POST_T_: 4.54°, *p* < 0.001, *d* = 2.568) and the thoracic spine (PRE_S_: 5.45°, POST_S_: 5.93°, *p* = 0.009, *d* = 0.863; PRE_F_: 12.82°, POST_F_: 14.89°, *p* < 0.001, *d* = 2.989; PRE_T_: 18.71°, POST_T_: 22.51°, *p* < 0.001, *d* = 1.728), showed significantly increased RoM with a high effect size in all three planes, except for the lumbar spine in the sagittal plane. Generally speaking, runners showed a tendency toward more joint motion especially in the sagittal plane. The RoM of the CoM increased significantly in the medio-lateral direction (PRE_medio−lateral_: 4.60°, POST_medio−lateral_: 5.11°, *p* = 0.039, *d* = 0.641), but decreased in the vertical direction (PRE_vertical_: 61.85°, POST_vertical_: 60.11°, *p* = 0.043, *d* = 0.627) with medium effect sizes. This means that runners moved more from side-to-side but less up-and-down.

**Table 2:** Range of motion of joints in degrees (°) and of the CoM in mm are shown as mean ± standard deviation for stance and flight phases separately.

	**PRE**	**POST**	***p***	***d***
***Stance phase***				
**Ankle**—**S [°]**	**51.15** **±** **4.38**	**53.55** **±** **4.37**	**<** **0.001**	**1.230**
Ankle—F [°]	17.32 ± 5.31	17.53 ± 5.36	0.568	0.163
Ankle—T [°]	11.11 ± 2.21	10.61 ± 2.41	0.363	0.262
**Knee**—**S [°]**	**37.81** **±** **5.23**	**40.97** **±** **6.12**	**<** **0.001**	**1.451**
Knee—F [°]	4.54 ± 3.54	4.78 ± 3.52	0.580	0.158
Knee—T [°]	7.16 ± 2.68	7.12 ± 3.35	0.953	0.017
**Hip**—**S [°]**	**53.33** **±** **5.53**	**56.87** **±** **6.24**	**<** **0.001**	**2.200**
**Hip**—**F [°]**	**17.10** **±** **3.60**	**18.82** **±** **3.58**	**<** **0.001**	**1.282**
**Hip**—**T [°]**	**9.39** **±** **5.05**	**11.86** **±** **5.35**	**<** **0.001**	**1.442**
Lumbar Spine—S [°]	12.13 ± 1.98	12.86 ± 2.47	0.088	0.514
**Lumbar Spine**—**F [°]**	**8.10** **±** **0.86**	**10.05** **±** **1.12**	**<** **0.001**	**1.513**
**Lumbar Spine**—**T [°]**	**3.78** **±** **0.54**	**4.54** **±** **0.68**	**<** **0.001**	**2.568**
**Thoracic Spine**—**S [°]**	**5.45** **±** **0.78**	**5.93** **±** **1.01**	**0.009**	**0.863**
**Thoracic Spine**—**F [°]**	**12.82** **±** **1.25**	**14.89** **±** **1.34**	**<** **0.001**	**2.989**
**Thoracic Spine**—**T [°]**	**18.71** **±** **4.10**	**22.51** **±** **22.51**	**<** **0.001**	**1.728**
COM ant-post [mm]	13.42 ± 1.62	14.14 ± 2.66	0.213	0.365
**COM med-lat [mm]**	**4.60** **±** **1.36**	**5.11** **±** **1.61**	**0.039**	**0.641**
**COM vertical [mm]**	**61.85** **±** **6.87**	**60.11** **±** **6.25**	**0.043**	**0.627**
***Flight phase***				
Ankle—S [°]	13.03 ± 4.17	11.44 ± 4.42	0.059	0.579
Ankle—F [°]	5.17 ± 3.08	5.67 ± 2.44	0.223	0.356
Ankle—T [°]	6.54 ± 3.00	6.39 ± 3.35	0.751	0.090
Knee—S [°]	99.52 ± 10.62	96.65 ± 11.63	0.057	0.583
Knee—F [°]	7.44 ± 3.96	8.16 ± 4.21	0.224	0.355
Knee—T [°]	11.48 ± 8.00	13.12 ± 6.55	0.065	0.564
**Hip**—**S [°]**	**22.96** **±** **6.14**	**20.75** **±** **5.21**	**0.001**	**1.155**
Hip—F [°]	8.85 ± 2.20	8.91 ± 1.43	0.877	0.044
Hip—T [°]	10.55 ± 4.22	10.94 ± 4.87	0.524	0.182
Lumbar spine—S [°]	11.03 ± 2.40	11.19 ± 2.36	0.584	0.156
Lumbar spine—F [°]	4.68 ± 1.33	4.33 ± 1.26	0.190	0.385
**Lumbar spine**—**T [°]**	**1.03** **±** **0.45**	**1.28** **±** **0.47**	**<** **0.001**	**1.210**
Thoracic spine—S [°]	4.75 ± 0.97	4.94 ± 1.01	0.117	0.468
Thoracic spine—F [°]	1.99 ± 0.72	2.12 ± 1.11	0.424	0.230
**Thoracic spine**—**T [°]**	**9.85** **±** **3.25**	**10.56** **±** **3.31**	**0.025**	**0.710**
COM ant-post [mm]	13.71 ± 2.78	12.94 ± 3.24	0.163	0.412
COM med-lat [mm]	8.60 ± 3.10	8.15 ± 2.34	0.428	0.227
**COM vertical [mm]**	**51.88** **±** **14.76**	**46.92** **±** **11.76**	**0.002**	**1.075**

*p-values as calculated by the dependent t-test and Cohen's d as effect sizes are also given. Significant differences (p < 0.05) are highlighted in bold. Cohen's d effect sizes of <0.50, 0.5–0.8, and >0.8 indicate small, medium, and large effects, respectively. S, F, and T signifies the sagittal, the frontal, and the transversal plane, respectively*.

In the flight phase, a smaller number of significant changes were detected compared to the stance phase. The RoM of the hip joint decreased significantly in the sagittal plane with a high effect size (PRE: 22.96°, POST: 20.75°, *p* = 0.001, *d* = 1.155), whereas those of the lumbar (PRE: 1.03°, POST: 1.28°, *p* < 0.001, *d* = 1.210) and the thoracic (PRE: 9.85°, POST°: 10.56, *p* = 0.025, *d* = 0.710) spine increased in the transverse plane. The effect sizes were high and medium, respectively, which means that upper body rotation increased. The RoM of the CoM decreased in the vertical direction with a high effect size (PRE: 51.88°, POST: 46.92°, *p* = 0.002, *d* = 1.075) but no significant changes were detected in the other planes (Table 2), which means that runners moved less up-and-down during flight.

In summary, the results revealed predominantly greater motion in the sagittal plane for the lower limbs and increased upper body motion especially in the transverse plane. Furthermore, the CoM showed less up-and-down-movement.

### Time Series Analyses of Joint and CoM Movements

**Figure 1:**
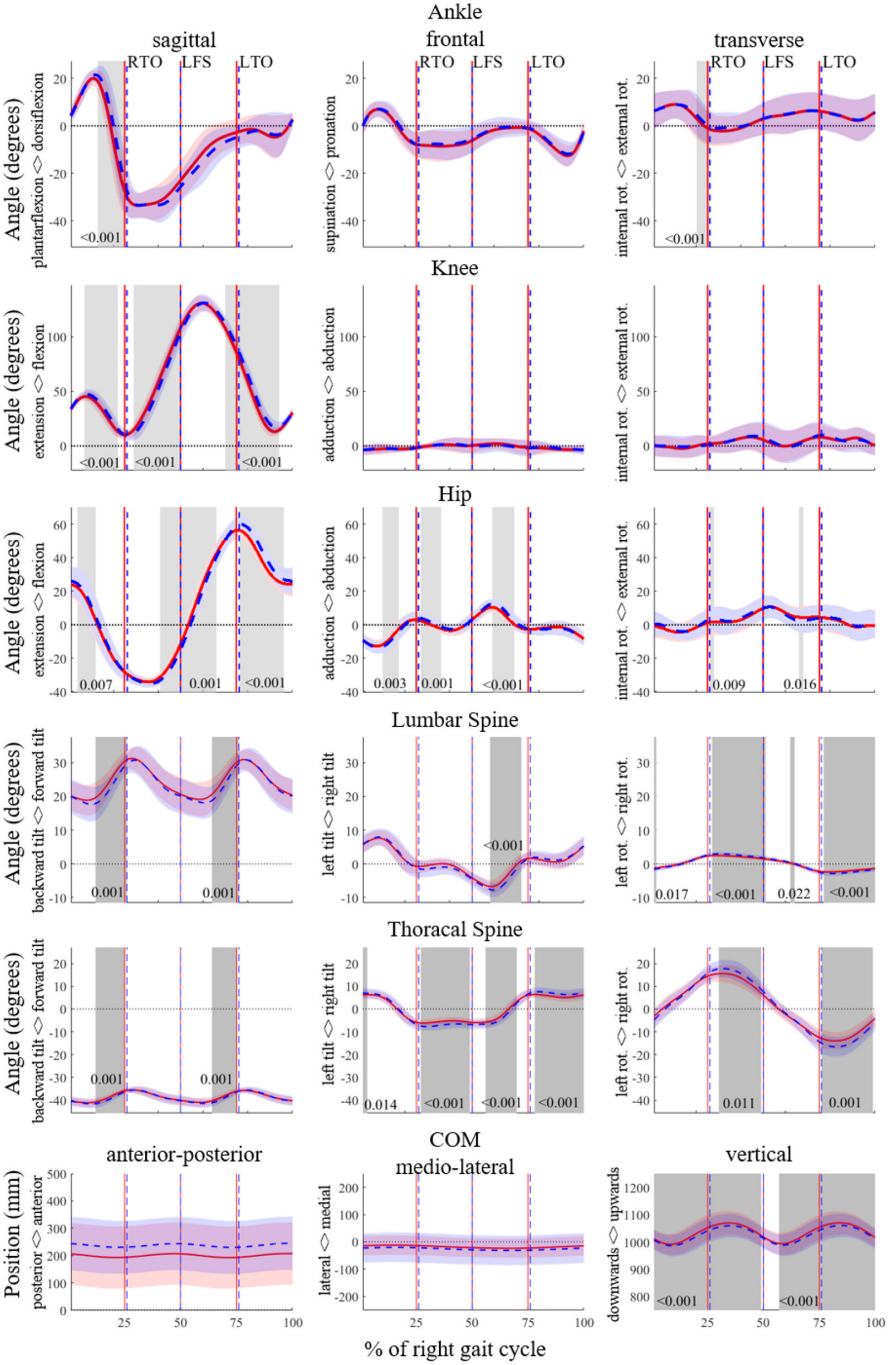
SPM analyses for the angles of the ankle, knee, hip, lumbar spine and thoracic spine in degrees, and of the trajectory of the center of mass (CoM) in mm for the entire running gait cycle of the right leg (from right foot strike to right foot strike) in 3D. The PRE and POST time series data are shown in red and blue, respectively. Significant differences (*p* < 0.05) are highlighted with gray areas and corresponding *p*-values are given. RTO signifies right toe off; LFS, left foot strike; LTO, left toe-off.

To prevent any over-simplification, the joint angle data were further analyzed by means of SPM. The trajectories of five joints as well as the CoM in all three planes are represented in Figure 1.

The SPM analysis (Figure 1) revealed an increase in dorsiflexion and external rotation prior to right toe off.

The knee joint showed more flexion particularly during late swing and during stance, whereas it was more extended during early swing in the POST. In the remaining planes, there were no significant differences.

The hip joint was less flexed during early and mid-swing, and more flexed during stance and late swing, in the POST. There were several significant differences between the PRE and the POST in the frontal plane of the hip joint. The hip joint was more adducted in the middle of the right stance phase and more abducted in the beginning of the right flight phase. Contrarily, it was more abducted in during mid swing.

The hip joint was less flexed around right foot strike, and more flexed after right toe-off, in the POST. There were several significant differences between the PRE and the POST in the frontal plane of the hip joint. The hip joint was more abducted in the middle of the right stance phase and in the beginning of the right flight phase. Contrarily, it was more adducted in the middle of the left stance phase as well as in the middle of the left flight phase.

The two joints representing trunk movement, in the lumbar and in the thoracic spine, showed less flexion in the sagittal plane, indicating a predominantly increased backwards tilt of the trunk in the POST. In the frontal plane, both the lumbar and the thoracic spine were more tilted to the left before left toe-off. After left toe-off, these areas were more tilted to the right and after right toe-off the thoracic spine was more tilted to the left. In the transverse plane, runners rotated to the right after left toe-off and rotated to the left after right toe-off. This occurred at both the lumbar and the thoracic spine joints, which overall indicates an increased rotation in the upper body.

During almost the entire gait cycle, the position of the CoM was lower in the POST compared to the PRE. In the remaining two directions, anterio-posterior and medio-lateral, there were not any significant changes.

## Discussion

This study is one of the first to investigate the effects of fatigue on expert runners during an exhaustive middle-distance run. The analysis was performed in 3D and entire time series were considered in the analysis by means of SPM. The results indicated that fatigue affects the spatiotemporal parameters, stiffness, CoM trajectories and joint kinematics throughout the gait cycle.

### Spatiotemporal Parameters and Their Variability

Between the PRE and POST, stride frequency fluctuated between 1.53 and 1.54 Hz (~92 strides per min). Since the speed was fixed during the fatigue protocol and the stride frequency did not change, the step length had to remain unchanged because speed is the multiplication of stride frequency with stride length. Since stride frequency did not change from PRE to POST, one could assume that trained runners choose a stride frequency and a step length associated with the lowest energy cost and try to keep them up (Williams and Cavanagh, 1987; Hunter and Smith, 2007). The stride frequency chosen by the athletes in the present study (~92 strides per min) was slightly higher than reported by Hunter and Smith (~86–87 strides per min) who analyzed changes with fatigue during a 1 h high-intensity run. This increase might be due to the higher running velocity (Fletcher and Macintosh, 2017). Even though stride frequency was the same in PRE and POST, contact time increased which was compensated by a decreased flight time.

### Vertical and Leg Stiffness and Their Variability

The results show that fatigued runners have a decreased leg and vertical stiffness in the POST, which leads to a longer contact time and shorter flight times. These results are in line with other studies (Dutto and Smith, 2002; Rabita et al., 2011, 2013; García-Pinillos et al., 2020). These decreases in stiffness may be explained by the reduced effectiveness of the stretch-shortening cycle and may possibly increase energy cost, which ultimately would decrease running performance (Hayes and Caplan, 2012; Pappas et al., 2014). The CV of both vertical and leg stiffness decreased with fatigue, which means that stiffness varied more from stride to stride in PRE compared to POST. In a study investigating relationships between coordinative variability and overuse injury (Hamill et al., 2012), a higher variability of a coordinative structure was related to a healthier state of athletes. However, a causal relationship between injury and variability was not yet found. Dutto and Smith (2002) also reported that the relationship between injury mechanisms and shifts in stiffness remained unclear.

### Analyses of Range of Motion

Increases in RoM were observed, mainly during the stance phase, which was also reported by Maas et al. (2018). In the ankle, knee, and hip joints, RoM in the sagittal plane increased with fatigue. Since the running speed was fixed by the treadmill, the horizontal mechanical power that each runner had to generate remained unchanged during the entire run. Accordingly, it may be assumed that a tradeoff between mechanical torque and angular displacement has been maintained during the run (Günther and Blickhan, 2002). Consequently, increased angular displacement, which manifests itself as increases in RoM in this case, may be explained by decreased torques at joints, probably due to decreased muscle forces occurring with fatigue (Hanon et al., 2005).

At the hip, the lumbar spine and the thoracic spine, the RoM increased in the frontal and transverse planes. These changes are possibly due to a fatigued core musculature causing difficulties in stabilizing the trunk (Koblbauer et al., 2014), and may be considered to be counterproductive since they do not produce any effective contribution to forward propulsion. On the other hand, increased upper body motion may also be a result of motor control system which tries to compensate increased lower body angular moment by increasing the upper body moment in the reverse direction (For more details see Section “Time Series Analyses of Joint and CoM Movements”). During stance, the CoM showed more movement in the medio-lateral direction and less movement in the vertical direction; this is also in line with the decreased stiffness discussed earlier in Section “Spatiotemporal Parameters and Their Variability.”

### Time Series Analyses of Joint and CoM Movements

The SPM showed that the ankle was less plantarflexed during the second half of stance. This is in accordance with Mizrahi et al. (2000), who found a decreased activity of the tibialis anterior and hypothesized that this led to a pendant toe. The difference in both knee and hip flexion looks like a time shift in the signal: in the POST, the knee flexion curve is behind the PRE curve, which might be caused by the longer stance phase. There was an increased level of movement in the upper body in the POST. Runners leaned more to the side, which is in accordance with the increased medio-lateral CoM movement during stance (for more details see Section “Vertical and Leg Stiffness and Their Variability”). Additionally, an increased upper body rotation was detected, which means there was an increase in movements which do not support forward propulsion. This was probably due to a decrease in trunk stability and possibly led to a decrease in running efficiency.

The SPM showed that many joint movements are affected, not only around initial contact and toe off but also in other phases of the running gait cycle. This finding is an indicator that the studies whose results are limited to discrete parameters may be missing some important aspects due to over-simplified treatments, as also mentioned by Pataky et al. (2013).

The significant changes between PRE and POST in the lower body mainly occurred in the sagittal plane, whereas the changes in the upper body were distributed in all three planes. Sagittal plane dominance within the changes in the lower body movements can be explained by the fact that forward propulsion is mainly associated with the extensions of hip, knee, and ankle joints. Increased level of lower body joint extensions leads to an increased lower body angular moment in vertical direction (i.e., moment due to rotation around the axis parallel to the direction of gravity). These increased rotational moments are counteracted by increased upper body moments around the this same axis, which is predominantly done by increasing upper body rotation (Hinrichs, 1987). Ultimately, the total moment of the body around the vertical direction approaches zero, so that the runners can sustain an optimum level of horizontal speed. Significant differences in CoM trajectories were only seen in the vertical direction, indicating that the angular moments in the lower and upper body were balanced such that CoM trajectories related to rotation in vertical direction remained unchanged. These findings may be transferred into practical usage as an indicator for the importance of functional core training. A properly functioning tradeoff mechanism between upper and lower body would optimize the horizontal speed, therefore the performance of the runners as well (Hinrichs, 1987). Any weakness or lack of sufficient coordination in the core muscles may potentially decrease the movement efficiency or increase the injury risk. Main focus of a proper core training should therefore be on the training of movements and positions, rather than just single muscles without considering their synergic behaviors within the complete body (Fredericson and Moore, 2005).

### Limitations and Outlook

There are some limitations of the present study that need to be mentioned. First, the use of a treadmill ensured a constant speed and thus enabled investigation of the effects of fatigue in isolation. However, one has to keep in mind that varying speed is a strategy which would be employed by runners when running overground. Besides, it should be noted that although the parameters estimated during treadmill running are comparable to those measured during overground running, they are not equivalent (Van Hooren et al., 2020). Since all participants underwent standardized treadmill familiarization, we can assume that participants had a stable running style. Second, the sample size could have been larger, although it is not easy to recruit a large sample of high-level runners. By using the results found in this exploratory study, subsequent studies may be able to formulate targeted hypotheses concerning the effects of fatigue on running performance or risk of injury. Third, participants of this study were chosen based on their 10 km performance, whereas fatigue protocol was considerably shorter (1.34 ± 0.27 km). This contrast may be considered as a limiting factor. However, even if it would have been preferable to select runners based on their 1,500 or 3,000 m performance, the goal of this study was to analyze fatigue-related changes during a middle-distance run of experienced runners.

## Conclusion

Despite the number of studies conducted, there is still no clear consensus on how running patterns change in a fatigued state. Compared to long-distance running, middle-distance running has been less frequently studied until now. In this study, the fatigue changes in expert runners during a middle-distance run were investigated in a highly standardized laboratory study by analyzing not only discrete parameters but also time series in 3D. Ultimately, an extensive picture of running in a fatigued state was presented.

The key findings from this study highlight that expert runners increase stance time and decrease time of flight, but keep both the step frequency and the step length constant. Concerning kinematics, increased upper body movements became apparent with fatigue, which may be transferred into the field as an indicator for the importance of functional core training (e.g., total body trainings focusing on core strength) in middle-distance runners. In the fatigued state runners increased their stance time, which led to increased lower body angular moments. These moments were counteracted by increased upper body rotation. The presented results may be used in future research or for practical uptake, particularly when designing training programs (e.g., integrating proper kind of functional core training).

## Data Availability Statement

The data analyzed in this study is subject to the following licenses/restrictions: The raw data supporting the conclusions of this article will be made available by the authors, without undue reservation. Requests to access these datasets should be directed to felix.moehler@hotmail.de.

## Ethics Statement

The studies involving human participants were reviewed and approved by the ethics committee of the Karlsruhe Institute of Technology. The patients/participants provided their written informed consent to participate in this study.

## Author Contributions

FM and TS were involved in the design of the study. FM carried out all data collection and data analysis. All authors were involved in the interpretation and discussion of the results. FM took the lead in writing the manuscript. All authors provided critical feedback and contributed to the final manuscript.

## Conflict of Interest

The authors declare that the research was conducted in the absence of any commercial or financial relationships that could be construed as a potential conflict of interest.
