# Corrigendum: Fatigue-Related Changes in Spatiotemporal Parameters, Joint Kinematics and Leg Stiffness in Expert Runners During a Middle-Distance Run

**DOI:** 10.3389/fspor.2022.872316

**Published:** 2022-03-25

**Authors:** Felix Möhler, Cagla Fadillioglu, Thorsten Stein

**Affiliations:** BioMotion Center, Institute of Sports and Sports Science (IfSS), Karlsruhe Institute of Technology, Karlsruhe, Germany

**Keywords:** locomotion, endurance, treadmill, middle-distance, SPM, range of motion, 3D movement analysis

In the original article, Figure 1 included the joint angles for the left leg instead of the joint angles for the right leg. Since the data were segmented from right heel strike to right heel strike, the gait events and the trajectories did not match. The corrected Figure 1 appears below.

**Figure 1:**
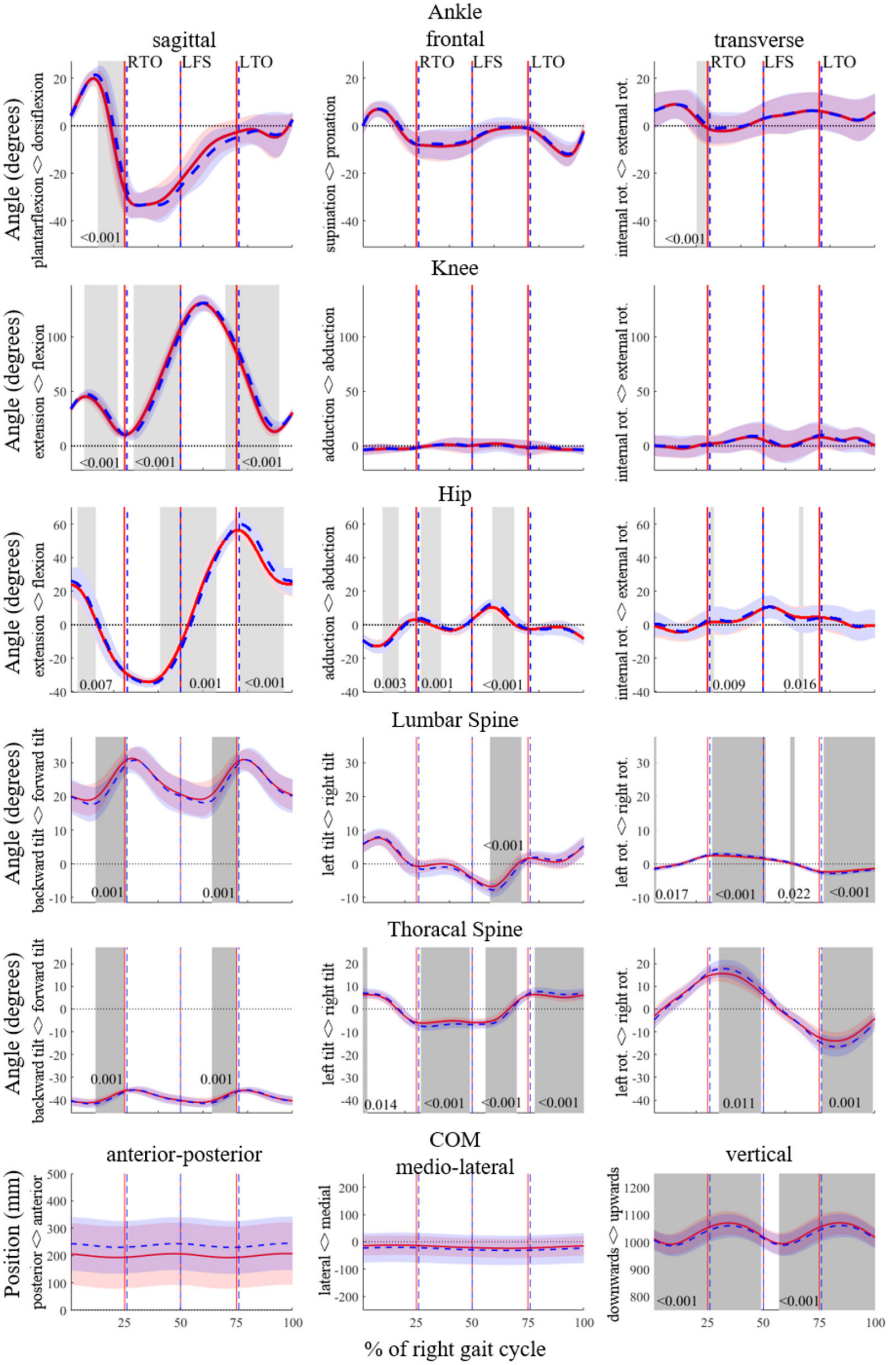
SPM analyses for the angles of the ankle, knee, hip, lumbar spine and thoracic spine in degrees, and of the trajectory of the center of mass (CoM) in mm for the entire running gait cycle of the right leg (from right foot strike to right foot strike) in 3D. The PRE and POST time series data are shown in red and blue, respectively. Significant differences (*p* < 0.05) are highlighted with gray areas and corresponding *p*-values are given. RTO signifies right toe off; LFS, left foot strike; LTO, left toe-off.

In the original article, there was an error in some formulations in the **Results** section, subsection **Time Series Analyses of Joint and CoM Movements** and **Discussion** section, subsection **Time Series Analyses of Joint and CoM Movements**, paragraph 1. The corrections in **Results** section and **Discussion** section appear below.

**Results, Time Series Analyses of Joint and CoM Movements**, paragraph two

Instead of “The SPM analysis (Figure 1) revealed a significantly higher plantarflexion of the ankle around right foot strike in the POST, as well as an increase in dorsiflexion and pronation prior to right foot strike. In the flight phase, the ankle was less plantarflexed and less supinated in the POST” the text has been corrected to “The SPM analysis (Figure 1) revealed an increase in dorsiflexion and external rotation prior to right toe off.”

**Results, Time Series Analyses of Joint and CoM Movements**, paragraph three

Instead of “The knee joint showed more flexion particularly during swing and around right toe-off, whereas it was less flexed before the right foot strike in the POST. In the remaining planes, there were no significant differences except for a change with a short duration in the transverse plane” the text has been corrected to “The knee joint showed more flexion particularly during late swing and during stance, whereas it was more extended during early swing in the POST. In the remaining planes, there were no significant differences.”

**Results, Time Series Analyses of Joint and CoM Movements**, paragraph four

Instead of “The hip joint was less flexed around right foot strike, and more flexed after right toe-off, in the POST. There were several significant differences between the PRE and the POST in the frontal plane of the hip joint. The hip joint was more abducted in the middle of the right stance phase and in the beginning of the right flight phase. Contrarily, it was more adducted in the middle of the left stance phase as well as in the middle of the left flight phase” the text has been corrected to “The hip joint was less flexed during early and mid-swing, and more flexed during stance and late swing, in the POST. There were several significant differences between the PRE and the POST in the frontal plane of the hip joint. The hip joint was more adducted in the middle of the right stance phase and more abducted in the beginning of the right flight phase. Contrarily, it was more abducted in during mid swing.”

**Discussion, Time Series Analyses of Joint and CoM Movements**, paragraph one

Instead of “The SPM showed that the ankle was less plantarflexed and supinated during flight.” the text has been corrected to “The SPM showed that the ankle was less plantarflexed during the second half of stance.”

The authors apologize for these errors and state that this does not change the scientific conclusions of the article in any way. The original article has been updated.

## Publisher's Note

All claims expressed in this article are solely those of the authors and do not necessarily represent those of their affiliated organizations, or those of the publisher, the editors and the reviewers. Any product that may be evaluated in this article, or claim that may be made by its manufacturer, is not guaranteed or endorsed by the publisher.

